# Supplementary material for: Glucosinolate Biosynthetic Genes of Cabbage: Genome-Wide Identification, Evolution, and Expression Analysis
Source: Genes (Basel). 2023 Feb 13;14(2):476. doi: 10.3390/genes14020476 (PMC9956868; doi:10.3390/genes14020476)
Supplement: Supplementary file 1 [file genes-14-00476-s001.zip › Table S1.pdf]

Table S1 Glucosinolate biosynthetic genes identified in cabbage

Enzyme	*Arabidopsis thaliana*	*B. oleracea*			
		Syntenic Orthologs			Non-Syntenic Orthologs
		LF	MF1	MF2	
IQD1-1	*AT3G09710*	-	*BolC01g052640.2J（IQD1-1-1）*	*BolC03g039550.2J（IQD1-1-2）*	-
MYB28	*AT5G61420*	*BolC07g043460.2J（MYB28-1）*	*BolC02g060440.2J（MYB28-2）*	*BolC09g007690.2J（MYB28-3）*	-
MYB34	*AT5G60890*	*BolC07g043000.2J（MYB34-1）*	*BolC02g060010.2J（MYB34-2）*	*BolC09g007420.2J（MYB34-3）*	*BolC02g060030.2J*
MYB51	*AT1G18570*	*BolC05g016180.2J（MYB51-1）*	*BolC08g027110.2J（MYB51-2）*	*BolC08g050820.2J（MYB51-3）*	-
MYB76	*AT5G07700*	*BolC09g064700.2J（MYB76-1）*	*BolC03g003550.2J（MYB76-2）*	-	-
MYB122	*AT1G74080*	*BolC06g045510.2J（MYB122-1）*	*BolC02g030750.2J（MYB122-2）*	-	-
MYB115	*AT5G40360*	*BolC04g045760.2J（MYB115）*	-	-	-
MYB118	*AT3G27785*	*BolC02g053640.2J(MYB118-1)*	*BolC07g035480.2J(MYB118-2)*	-	-
BCAT-4	*AT3G19710*	*BolC05g044530.2J（BCAT-4-1）*	-	*BolC03g046400.2JBol018130（BCAT-4-2）*	-
BAT5	*AT4G12030*	*BolC09g031400.2J（BAT5-1）*	*BolC03g032630.2J（BAT5-2）*	-	-
MAM1	*AT5G23010*	-		-	-
MAM3	*AT5G23020*	*BolC07g042910.2J（MAM3-1）*	*BolC02g059940.2J（MAM3-2）*	-	*BolC07g042920.2J*
IPMI LSU1	*AT4G13430*	-	-	-	-
IPMI SSU2	*AT2G43100*	-	-	-	-
IPMI SSU3	*AT3G58990*	-	-	-	-
IPMDH1	*AT5G14200*	-	-	*BolC02g006120.2J（IPMDH1）*	-
IPMDH3	*AT1G31180*	-	-	-	-
BCAT-3	*AT3G49680*	*BolC08g033700.2J（BCAT-3-1）*	*BolC01g029190.2J（BCAT-3-2）*	-	-
CYP79F1	*AT1G16410*	-	-	-	-
CYP79F2	*AT1G16400*	*BolC05g014000.2J（CYP79F2-1）*	-	-	-
CYP79A2	*AT5G05260*	*BolC09g066710.2J（CYP79A2-1）*	-	*BolC02g002250.2J（CYP79A2-3）*	-
CYP83B1	*AT4G31500*	-	-	-	-
CYP79B2	*AT4G39950*	*BolC01g000930.2J（CYP79B2-1）*	*BolC07g060340.2J（CYP79B2-2）*	*BolC03g073270.2J（CYP79B2-3）*	-
CYP79B3	*AT2G22330*	-	*BolC04g049480.2J（CYP79B3）*	-	-
CYP83A1	*AT4G13770*	-	*BolC04g041440.2J（CYP83A1）*	-	-
CYP83B1	*AT4G31500*	-	-	-	-
GSTF9	*AT2G30860*	-	-	*BolC04g057100.2J（GSTF9-2）*	-
GSTF10	*AT2G30870*	*BolC03g018460.2J（GSTF10-1）*	-	*BolC04g057100.2J（GSTF10-2）*	-
GSTF11	*AT3G03190*	-	-	-	-
GSTU20	*AT1G78370*	*BolC06g049340.2J（GSTU20-1）*	*BolC02g034520.2J（GSTU20-2）*	*BolC06g029240.2J（GSTU20-3）*	*BolC06g049350.2J/BolC06g049360.2J*
GGP1	*AT4G30530*	*BolC01g008500.2J（GGP1-1）*	*BolC07g055380.2J（GGP1-2）*	*BolC03g082130.2J（GGP1-3）*	*BolC03g082140.2J*
SUR1	*AT2G20610*	*BolC07g000300.2J（SUR1-1）*	*BolC09g014020.2J（SUR1-2）*	*BolC08g049710.2J（SUR1-3）*	*BolC07g000320.2J*
UGT74B1	*AT1G24100*	*BolC05g022270.2J（UGT74B1-1）*	-	-	-
UGT74C1	*AT2G31790*	*BolC04g017450.2J（UGT74C1-1）*	*BolC04g058020.2J（UGT74C1-2）*		-
ST5a	*AT1G74100*	-	-	-	-
ST5b	*AT1G74090*	*BolC06g045520.2J（ST5b-1）*	*BolC02g030760.2J（ST5b-2）*	*BolC06g031930.2J（ST5b-3）*	*BolC06g045530.2J*
ST5c	*AT1G18590*	*BolC05g016200.2J（ST5c）*	-	-	-
FMOGS-OX1	*AT1G65860*	-	-	-	-
FMOGS-OX2	*AT1G62540*	-	-	-	-
FMOGS-OX3	*AT1G62560*	-	-	-	-
FMOGS-OX4	*AT1G62570*	-	-	-	-
FMOGS-OX5	*AT1G12140*	-	*BolC08g023820.2J（FMOGS-OX5-1）*	*BolC08g054910.2J（FMOGS-OX5-2）*	-
AOP1	*AT4G03070*	-	*BolC03g033940.2J（AOP1-2）*	*BolC02g038710.2J（AOP1-3）*	-
AOP2	*AT4G03060*	*BolC09g002350.2J（AOP2）*	-	-	-
AOP3	*AT4G03050*	-	-	-	-
GSL-OH	*AT2G25450*	-	-	-	-
CYP81F2	*AT5G57220*	*BolC09g045820.2J（CYP81F2-1）*		*BolC02g015480.2J（CYP81F2-3）*	-
BZO1p1	*AT1G65880*	*BolC06g037140.2J（BZO1p1-1）*	*BolC02g022010.2J（BZO1p1-2）*	-	-
APK1	*AT2G14750*	-	*BolC09g011620.2J（APK1-1）*	*BolC03g051190.2J（APK1-2）*	-
APK2	*AT4G39940*	*BolC01g000920.2J（APK2-1）*	*BolC07g059810.2J（APK2-2）*	*BolC03g073230.2J（APK2-3）*	-
GSH1/PAD2	*AT4G23100*	*BolC01g016760.2J(GSH1/PAD2-1)*	*BolC07g050320.2J(GSH1/PAD2-2)*	*BolC03g078480.2J(GSH1/PAD2-3)*	*BolC07g050340.2J*
CHY1	*AT5G65940*	-	*BolC02g063610.2J(CHY1-1)*	*BolC09g011290.2J(CHY1-2)*	-

AAO4	*AT1G04580*	*BolC05g002740.2J(AAO4)*	-	-	-
BCAT-6	*AT1G50110*	*BolC06g004630.2J(BCAT-6-1)*	*BolC03g082990.2J(BCAT-6-2)*	-	*BolC03g083000.2J*
CYTB5C	*AT2G46650*	*BolC04g001960.2J（CYTB5C-1）*		-	-
GSTU13	*AT1G27130*	*BolC05g022730.2J（GSTU13-1）*	*BolC07g019530.2J（GSTU13-2）*	-	-
CYP81F1	*AT4G37430*	*BolC01g001620.2J（CYP81F1-1）*		-	*BolC01g001630.2J*
CYP81F2	*AT4G37400*	-	*BolC03g073890.2J（CYP81F2-2）*	-	-
CYP81F4	*AT4G37410*	*BolC01g001680.2J（CYP81F4-1）*	*BolC03g073910.2J（CYP81F4-2）*	-	-
IGMT1	*AT1G21100*	-	-	-	-
IGMT2	*AT1G21120*	*BolC07g021580.2J（IGMT2-1）*	*BolC08g029030.2J（IGMT2-2）*	*BolC07g021570.2J（IGMT2-3）*	*BolC08g029040.2J/BolC07g021580.2J/BolC07g021590.2J*
IGMT5	*AT1G76790*	*BolC06g048200.2J（IGMT5-1）*	*BolC06g030210.2J（IGMT5-2）*	-	-
TSB1	*AT5G54810*	*BolC09g042250.2J（TSB1-1）*	*BolC02g017380.2J（TSB1-2）*	-	-
ASA1	*AT5G05730*	*BolC03g002500.2J（ASA1-1）*	*BolC09g066270.2J（ASA1-2）*	-	-
GSH2	*AT5G27380*	*BolC09g005320.2J（GSH2-1）*	*BolC07g039320.2J（GSH2-2）*	*BolC02g057030.2J（GSH2-3）*	-
APS1	*AT3G22890*	*BolC03g048340.2J（APS1-1）*	*BolC05g039450.2J（APS1-2）*	-	-
APS3	*AT4G14680*	*BolC01g027020.2J（APS3）*	-	-	-
APR1	*AT4G04610*	*BolC09g030170.2J（APR1-1）*	*BolC02g039130.2J（APR1-2）*	-	-
APR2	*AT1G62180*	*BolC04g028240.2J（APR2）*	-	-	-
APR3	*AT4G21990*	*BolC07g049530.2J（APR3-1）*	*BolC05g063700.2J（APR3-2）*		-
OASA1	*AT4G14880*	*BolC08g014200.2J（OASA1-1）*	*BolC01g026690.2J（OASA1-2）*	-	-
SCPL17	*AT3G12203*	*BolC01g050610.2J（SCPL17-1）*	*BolC03g041700.2J（SCPL17-2）*		*BolC01g050620.2J/BolC01g050630.2J/BolC03g041710.2J*
TGG1	*AT5G26000*	-	-		-
TGG2	*AT5G25980*	-	-	-	-
TGG4	*AT1G47600*	-	-	-	-
TGG5	*AT1G51470*	*BolC06g012830.2J （TGG5-1）*	*BolC03g083700.2J（TGG5-2）*		-
ESP	*AT1G54040*	*BolC02g044590.2J（ESP-1）*	*BolC07g026490.2J（ESP-2）*		-
NIT1	*AT3G44310*	-	*BolC02g007890.2J（NIT1-2）*	-	-
NIT2	*AT3G44300*	-	-	-	-
NIT4	*AT5G22300*	*BolC02g011540.2J（NIT4）*	-	-	-
NSP3	*AT3G16400*	*BolC01g046450.2J（NSP3-1）*	*BolC05g049830.2J（NSP3-2）*	*BolC03g044470.2J （NSP3-3）*	*BolC01g046430.2J/BolC01g046440.2J*
NSP2	*AT2G33070*	*BolC04g058490.2J（NSP2）*	-	-	-
NSP4	*AT3G16410*	-	-	-	-
NSP5	*AT5G48180*	*BolC07g037340.2J（NSP5-1）*	*BolC02g055310.2J（NSP5-2）*	-	-
PYK10	*AT3G09260*	*BolC03g039780.2J（PYK10-1）*	*BolC01g052950.2J（PYK10-2）*	*BolC08g011610.2J（PYK10-3）*	-
ESM1	*AT3G14210*	*BolC05g052270.2J（ESM1-1）*	*BolC01g048540.2J（ESM1-2）*	*BolC03g043000.2J （ESM1-3）*	-
PCS1	*AT5G44070*	*BolC07g025820.2J（PCS1-1）*	*BolC02g044130.2J（PCS1-2）*	*BolC09g024110.2J（PCS1-3）*	-
BGLU28	*AT2G44460*	*BolC04g066960.2J（BGLU28-1）*	*BolC04g005920.2J（BGLU28-2）*	-	*BolC04g066950.2J/BolC04g066980.2J*
BGLU30	*AT3G60140*	*BolC04g032580.2J（BGLU30-1）*	*BolC06g025390.2J（BGLU30-2）*	*BolC04g032630.2J（BGLU30-3）*	*BolC08g043460.2J/BolC08g043470.2J*
MYC2	*AT1G32640*	*BolC05g037770.2J（MYC2-1）*	*BolC08g011240.2J（MYC2-2）*	-	-
MYC3	*AT5G46760*	*BolC07g029330.2J（MYC3-1）*	*BolC09g026200.2J（MYC3-2）*	-	-
MYC4	*AT4G17880*	*BolC01g011310.2J（MYC4）*	-	-	-
SLIM1	*AT1G73730*	*BolC06g045150.2J（SLIM1-1）*	*BolC06g032080.2J（SLIM1-2）*	*BolC02g030530.2J（SLIM1-3）*	*BolC02g030550.2J*
OBP2	*AT1G07640*	*BolC08g003030.2J（OBP2-1）*	*BolC05g005480.2J（OBP2-2）*	*BolC08g057480.2J（OBP2-3）*	-
CAMTA3	*AT2G22300*	*BolC04g049460.2J（CAMTA3-1）*	*BolC08g048630.2J（CAMTA3-2）*		-
CCA1	*AT2G46830*	*BolC04g001800.2J（CCA1）*	-	-	-
HY5	*AT5G11260*	*BolC09g061470.2J（HY5-1）*	*BolC02g004670.2J（HY5-2）*	-	-
SD1	*AT5G48850*	*BolC07g037870.2J（SD1-1）*	*BolC09g004660.2J（SD1-2）*	*BolC02g055830.2J（SD1-3）*	-
SD2	*AT1G04770*	*BolC05g002890.2J（SD2-1）*	*BolC08g001460.2J（SD2-2）*		-
FRS12	*AT5G18960*	*BolC03g009880.2J（FRS12）*	-	-	-
MED5	*AT3G23590*	*BolC07g013940.2J（MED5-1）*	*BolC01g038710.2J（MED5-2）*	-	-
MED25/PFT1	*AT1G25540*	*BolC03g068250.2J（MED25/PFT1-1）*	*BolC05g023830.2J（MED25/PFT1-2）*	-	-
SULTR1;1	*AT4G08620*	-	-	-	-
SULTR1;2	*AT1G78000*	*BolC02g034190.2J（SULTR1;2-1）*	*BolC06g049010.2J（SULTR1;2-2）*	-	*BolC06g049010.2J*
GTR1	*AT3G47960*	*BolC03g061290.2J（GTR1-1）*	*BolC03g067640.2J（GTR1-2）*	*BolC03g067570.2J（GTR1-3）*	-
GTR2GTR2	*AT5G62680*	*BolC02g061160.2J（GTR2-1）*	*BolC03g058480.2J（GTR2-2）*	*BolC09g008180.2J（GTR2-3）*	-
