# Supplementary material for: Glucosinolate Biosynthetic Genes of Cabbage: Genome-Wide Identification, Evolution, and Expression Analysis
Source: Genes (Basel). 2023 Feb 13;14(2):476. doi: 10.3390/genes14020476 (PMC9956868; doi:10.3390/genes14020476)
Supplement: Supplementary file 1 [file genes-14-00476-s001.zip › Table S2.pdf]

Supplementary File S2: Expression of BoGBG genes in different organs of bud, callus, flower, leaf, silique and stem in homozygous cabbage line 02-12

Gene name	ID	Bud	Callus	Flower	Leaf	Silique	Stem
IQD1-1-1	*Bol023096*	1.76847	0.729348	0.393055	1.48845	0.308924	0.872013
MYB28-1	*Bol017019*	0.171866	0.378028	0	1.27614	0	3.05041
MYB28-3	*Bol036286*	6.78576	7.3319	79.8483	8.13375	2.139	61.7538
MYB29-1	*Bol043899*	0	0.994074	0.223216	0	0.67669	0.445696
MYB29-2	*Bol008849*	0	0	0	0.423956	0	0.844476
MYB34-3	*Bol036262*	0	0.821633	0	0	0.24858	0
MYB51-1	*Bol030761*	0	0.894667	0.0730527	0	0.221462	0
MYB51-2	*Bol013207*	0.219206	21.0541	0.0721778	0.795869	14.6603	1.29705
MYB76-1	*Bol043899*	0	0.994074	0.223216	0	0.67669	0.445696
MYB76-2	*Bol008849*	0	0	0	0.423956	0	0.844476
MYB122-1	*Bol026204*	0.895593	11.2449	0.294891	0.147801	19.9655	0.588809
BCAT-4-1	*Bol018130*	0.878848	18.0668	13.4894	15.3293	0.607334	327.745
BCAT-4-2	*Bol026690*	0.676037	2.67639	5.80965	8.85765	0.43855	80.8919
BAT5-1	*Bol020071*	0	0.953706	0.734235	0.858672	0.247318	4.88681
BAT5-2	*Bol030524*	0.115118	0.506415	1.25085	0.911905	0.11491	19.2995
MAM1-1	*Bol017071*	0	0.0649909	0	0	0	3.76779
MAM1-2	*Bol020644*	0	0	0	0	0	0
MAM3	*Bol036246*	0.994558	1.43034	0.982432	3.10591	3.89468	1.35804
BCAT-3-1	*Bol037342*	11.7286	13.876	6.20238	18.1241	7.7458	4.6149
BCAT-3-2	*Bol021961*	13.2218	27.8649	6.55905	10.266	8.95363	14.475
CYP79F1-1	*Bol038222*	1.03851	7.6834	4.94271	14.0225	0.0471196	154.368
CYP79A2-1	*Bol044048*	0.714558	4.35243	1.84606	11.9739	0.0548668	4.71594
CYP79A2-2	*Bol015432*	0	0	0	0	0.0489174	0
CYP79A2-3	*Bol024473*	0	0	0.116461	0	0.117685	0
CYP79B2-1	*Bol028852*	14.7512	21.6308	62.296	61.0167	1.75614	7.87423
CYP79B2-3	*Bol032767*	1.88092	2.06859	7.52485	0.838109	9.66924	3.10699
CYP83A1	*Bol040365*	17.4043	41.5432	91.8193	247.865	10.0452	728.643
CYP83B1-1	*Bol017993*	0	0.949807	0.10667	0	0.107791	0.106494
CYP83B1-2	*Bol033732*	0	0.254408	0	0	0	0
GSTF9	*Bol033376*	771.919	126.869	125.671	956.438	216.203	864.315
GSTF10	*Bol004624*	443.356	389.801	261.275	636.581	118.364	754.785
GSTU20-2	*Bol018963*	50.5076	47.6118	69.8066	30.2594	0.847334	84.3411
GSTU20-3	*Bol021560*	0.0604082	0.0664356	0.238687	0.239262	0	0.297866
GGP1-1	*Bol018073*	62.6166	112.6	157.514	37.0666	145.195	100.297
GGP1-2	*Bol033672*	60.2831	105.007	102.769	71.3411	127.045	81.0243
GGP1-3	*Bol012988*	11.3475	5.98344	6.29556	7.54209	5.58593	12.8769
SUR1-1	*Bol038764*	9.50947	14.9049	19.2922	5.05422	8.75717	20.7731
SUR1-3	*Bol045893*	29.5276	3.86142	4.54856	11.7408	6.95203	78.4466
UGT74B1-1	*Bol005786*	16.1955	17.8729	31.3892	36.4418	21.5736	89.9917
UGT74B1-2	*Bol007409*	0.41465	4.44645	0.170651	0	0.103434	0.545279
UGT74C1-1	*Bol014127*	5.50045	8.04613	30.9651	120.034	4.37109	437.903
ST5b-1	*Bol026200*	12.9584	28.8194	15.716	46.6201	10.9229	18.9559
ST5b-2	*Bol039395*	81.0128	74.882	43.2934	103.954	612.048	82.8835
ST5b-3	*Bol039973*	0	0	0	0.0827585	0	0
FMOGS-OX5-1	*Bol029100*	5.81587	1.55421	4.08054	3.76745	0.37979	9.64846
FMOGS-OX5-2	*Bol031350*	12.3616	62.6072	47.3238	10.6643	16.3112	31.0262
AOP1-1	*Bol030626*	0	0.424348	0	0	3.37008	0
CYP81F2-1	*Bol012237*	0.470834	15.4481	0	0	2.74157	0.154775
CYP81F2-2	*Bol014239*	0.0988053	16.0823	0	0	2.12048	1.16927
APK1-1	*Bol019336*	8.6397	34.7745	16.2769	9.1723	28.095	26.9662
APK1-2	*Bol042602*	13.3831	19.8649	27.5199	14.0314	3.05602	67.1969
APK2-1	*Bol028851*	0.334666	0.69011	0.371909	1.03557	0.167031	1.1964

APK2-2	*Bol018584*	8.43831	9.00732	30.8901	3.11284	2.3948	58.2517
APK2-3	*Bol032769*	0	0.192401	0	0	0.0873149	0.172527
GSH1/PAD2-3	*Bol010659*	7.48328	10.3801	9.38989	7.3764	9.79137	5.11934
AAO4	*Bol040907*	7.79317	5.31645	2.48888	1.91467	0.731118	9.36121
BCAT-6	*Bol015595*	14.0328	33.1023	4.08486	53.2981	28.1164	19.4546
CYTB5C-1	*Bol004913*	7.54972	17.7646	28.9635	21.0361	30.3196	69.2592
CYTB5C-2	*Bol021793*	35.9747	15.3087	50.1791	211.224	57.3833	155.815
GSTU13	*Bol004386*	333.534	31.4311	78.8777	499.844	94.238	156.65
CYP81F1	*Bol028913*	30.0669	22.5456	85.4365	0.628302	3.65413	29.1939
CYP81F2-1	*Bol028919*	0.341669	0.85888	1.35001	0	1.16932	2.16608
CYP81F2-2	*Bol032711*	0.196418	0.054004	0.0485058	0.0486227	0.0490158	0.0484257
CYP81F4-1	*Bol032712*	0.0486154	1.09587	0.336159	10.3265	2.57185	0.479433
CYP81F4-2	*Bol028918*						
IGMT1	*Bol007029*	1.31524	0.482156	0.192474	1.35057	25.1875	0.048039
IGMT2	*Bol007030*	0	50.6739	0	0.128883	1.23428	2.11795
IGMT5-1	*Bol027603*	24.4707	28.5763	11.8263	115.942	38.6753	35.2256
IGMT5-2	*Bol039840*	1.18407	6.29402	0.129959	11.4639	17.0723	0.90821
TSB1	*Bol038903*	48.967	76.3155	39.3685	102.34	108.108	66.8314
ASA1-1	*Bol044016*	18.836	15.6266	13.9143	15.813	21.8675	2.26138
ASA1-2	*Bol015425*	21.3478	24.3041	17.0441	11.544	32.0674	10.833
GSH2-1	*Bol032401*	19.8953	133.769	11.1628	11.3998	9.26771	13.8758
APS1-1	*Bol038597*	31.302	59.7482	33.3835	15.4449	29.6037	51.379
APS3	*Bol014728*	25.4523	69.2311	12.9849	34.1221	349.677	39.7683
APR1-1	*Bol012039*	21.705	22.2987	15.9496	5.81859	2.11374	20.4653
APR1-2	*Bol004599*	0	0	0	0	0	0
APR3-1	*Bol028479*	37.2033	109.753	25.95	20.3688	15.1027	58.4571
APR3-2	*Bol024279*	15.0103	120.924	22.5521	13.304	18.4933	39.2327
OASA1-1	*Bol028323*	63.0956	54.8256	69.7497	68.8127	30.3768	123.199
OASA1-2	*Bol014705*	103.872	146.46	222.547	115.979	64.5863	157.553
TGG2	*Bol028319*	0.124198	3.41476	0.122684	0.860857	117.558	0
TGG4-1	*Bol044760*	0	0	0	0	0	0
TGG4-2	*Bol044759*	0	0	0	0.00458925	0	0
ESP-1	*Bol024137*	0.0709445	4.75942	0.630716	1.47522	351.887	0
ESP-2	*Bol006380*	21.1513	64.2917	15.3088	299.122	1.1118	23.4039
ESP-3	*Bol039072*	72.7181	37.2171	240.513	11.0993	1.84123	233.539
NIT2-1	*Bol033384*	7.09203	108.598	61.2229	8.88133	7.00965	8.22823
NIT2-2	*Bol041291*	46.7935	572.408	691.758	80.0008	69.89	125.755
NIT2-3	*Bol021321*	427.825	736.142	3842.58	158.573	3.15902	411.507
NIT4	*Bol036051*	15.7473	70.0239	18.2489	0.877517	2.17751	26.4878
NSP2	*Bol034775*	5.02815	21.8496	1.09028	0.242869	19.0969	0.120943
PYK10-1	*Bol023070*	2.18482	102.324	2.34186	0.138088	108.812	0.0916859
ESM1	*Bol005067*	0.0627376	28.4269	0.185918	0.124244	74.4603	26.0474
PCS1-1	*Bol012856*	9.74188	42.3032	11.8553	8.05516	30.1754	8.71582
PCS1-2	*Bol024165*	0.986057	28.7106	0.340912	2.24568	37.2057	2.62555
BGLU30-1	*Bol045600*	1.62415	315.357	0.337757	0	0.810606	51.9495
MYB118	*Bol031189*	0.0616286	0	0.0608772	0	0	10.6967
MYC2-1	*Bol020888*	27.3207	21.0415	49.8719	33.7763	93.0975	101.903
MYC2-2	*Bol014189*	6.56231	12.736	12.4562	7.34732	137.602	21.3605
MYC3-1	*Bol008483*	23.8991	38.568	37.5559	75.0841	27.1798	65.8429
MYC4	*Bol019715*	5.09423	4.10417	4.0374	37.7732	8.45534	33.9983
SLIM1-1	*Bol026238*	9.78696	22.214	9.91447	8.37132	17.6263	9.07667
SLIM1-2	*Bol039991*	5.07898	7.14787	4.12419	3.75055	6.5306	4.5843
OBP2-1	*Bol023400*	5.39094	12.4986	3.52615	2.88543	7.70821	4.45429
OBP2-2	*Bol041144*	1.23111	0.796439	0.429211	0.215123	0.578299	0.642754
OBP2-3	*Bol006511*	0.703988	2.75282	1.93168	0.464721	1.71775	1.07995
CAMTA3-1	*Bol044752*	0.394902	6.97782	0.754168	1.64231	3.65281	2.0251
CCA1	*Bol004898*	8.38326	65.0807	40.7672	0.547021	2.23821	16.953

HY5-1	*Bol043589*	23.5909	41.315	87.7354	20.6498	5.83166	21.2961
HY5-2	*Bol024704*	14.5975	21.2391	19.8914	17.0486	4.40869	7.67799
SD1-1	*Bol032338*	0	81.1752	0.0785257	0.15743	0	0
SD1-2	*Bol043141*	1.77123	32.8556	1.55531	15.3177	78.7398	13.1983
SD1-3	*Bol033076*	1.34185	60.0441	0.414216	3.98606	0.251143	1.98495
SD2-1	*Bol040923*	24.4049	48.0108	16.4694	32.0611	5.54752	11.1203
SD2-2	*Bol011565*	2.85143	35.7867	4.30785	6.31126	1.92543	5.1278
MED5	*Bol037298*	6.44049	11.892	1.74475	2.49546	14.2123	2.48535
MED25/PFT1-1	*Bol024977*	9.01792	13.3873	11.3481	14.9413	12.0319	12.6208
SULTR1;2-1	*Bol018986*	3.77473	9.65911	8.19577	0	0.111918	0.0368569
GTR1-1	*Bol024088*	3.5092	5.61359	2.99373	4.2645	5.2543	4.48317
GTR1-2	*Bol024931*	16.6397	49.7586	13.8148	13.5342	276.076	24.4192
GTR1-3	*Bol018722*	2.02054	1.26357	2.19158	1.09843	1.14686	2.61774
GTR2-1	*Bol019440*	14.2528	103.2	38.1471	10.6044	1127.04	14.0165
